# Staff testing for COVID-19 via an online pre-registration form

**DOI:** 10.4102/sajid.v36i1.232

**Published:** 2021-01-22

**Authors:** Muhammad S. Moolla, Arifa Parker, Mohammed A. Parker, Sthembiso Sithole, Leila Amien, Rubeena Chiecktey, Tasneem Bawa, Abdurasiet Mowlana

**Affiliations:** 1 Department of Medicine, Faculty of Medicine and Health Services, Stellenbosch University and Tygerberg Hospital, Cape Town, South Africa

**Keywords:** COVID-19, SARS-CoV-2, healthcare workers, occupational health, staff coronavirus testing, outpatient coronavirus testing, workforce retention in pandemic, digital healthcare services

## Abstract

**Background:**

Healthcare workers are at increased risk of contracting severe acute respiratory syndrome coronavirus 2 (SARS-CoV-2) and potentially causing institutional outbreaks. Staff testing is critical in identifying and isolating infected individuals, whilst also reducing unnecessary workforce depletion. Tygerberg Hospital implemented an online pre-registration system to expedite staff and cluster testing. We aimed to identify specific presentations associated with a positive or negative result for SARS-CoV-2.

**Methods:**

A retrospective descriptive study design involving all clients making use of the hospital’s pre-registration system during May 2020.

**Results:**

Of 799 clients, most were young and females with few comorbidities. Nurses formed the largest staff contingent in the study, followed by administrative staff, doctors and general assistants. Doctors tested earlier compared with other staff (median: 1.5 vs. 4 days). The most frequent presenting symptoms included headache, sore throat, cough and myalgia. Amongst those testing positive (*n* = 105), fever, altered smell, altered taste sensation, and chills were the most common symptoms. Three or more symptoms were more predictive of a positive test, but 12/145 asymptomatic clients also tested positive.

**Conclusion:**

Staff coronavirus testing using an online pre-registration form is a viable and acceptable strategy. Whilst some presentations are less likely to be associated with SARS-CoV-2 infection, no symptom can completely exclude it. Staff testing should form part of a bundle of strategies to protect staff, including wearing masks, regular handwashing, buddy screening, physical distancing, availability of personal protective equipment and special dispensation for coronavirus disease 2019 (COVID-19)-related leave.

## Introduction

The emergence of severe acute respiratory syndrome coronavirus 2 (SARS-CoV-2) has placed severe pressure on an already overburdened healthcare system in South Africa. At Tygerberg Hospital (TBH), initially the designated coronavirus disease 2019 (COVID-19) hospital in the Western Cape, South Africa, mitigation strategies have included the establishment of separately staffed high-risk COVID-19 areas with clear signage, guidelines on the use of personal protective equipment (PPE), staff education and training, and the construction of new infrastructure for triage, testing and treatment with multi-departmental involvement.^1^

These measures aimed to meet the challenge of COVID-19 whilst protecting staff, but healthcare workers remain at increased risk of contracting SARS-CoV-2, potentially causing institutional outbreaks and spreading infection back to their communities.^2^ This can result in the closure of the entire department or hospital as has been reported in the media. Tygerberg Hospital has not been immune, with numerous confirmed positive cases and COVID-19-related deaths reported amongst staff.

Isolation of potentially SARS-CoV-2-infected healthcare workers is critical in preventing nosocomial spread. However, workforce depletion in the context of existing staff shortages weakens the fight against COVID-19. A balanced approach is required.^3^

The Occupational Health Department (OHD) developed a risk assessment tool to identify individuals at risk for severe disease and place staff in the most appropriate areas to work. A system was also implemented to screen and test staff members, both symptomatic and contacts of confirmed cases, that would allow for early return to work according to current national guidelines, mitigating against staff shortages whilst at the same time identifying and isolating infected individuals.^4^

The necessity of staff testing created additional workload for the Triage and Testing Centre (TATC) which, by late April 2020, was routinely processing over 200 tests per day. Smoothly processing staff members rapidly became more difficult as the number of cases presenting increased. To minimise waiting time and decrease the risk of cross-infection from traditional paper-based formats, an online pre-registration system for staff and clusters was implemented. The form (available at http://www.tbhcovid.co.za/register) was completed prior to arrival and included all relevant screening information. Once submitted, it generated an email to the clerks in the TATC, allowing a folder to be opened. On arrival, staff members in the TATC could retrieve the folder and test the individual in as little as 10 min.

We aimed to determine the reasons for testing and specific presentations associated with positive or negative results. This information could be used to refine future healthcare worker testing and isolation guidelines. We also aimed to identify specific sectors of staff where testing was delayed or deficient and for whom more targeted interventions may be required.

## Methods

We conducted a single-centre retrospective descriptive study involving all cases created via the pre-registration system from 01 to 31 May 2020. All cases were included as there were no exclusion criteria.

We collected self-reported information from all emails received over the study period, including date of registration, demographic information, symptoms, contact history, comorbidities and employment information. Data were exported from the inbox and algorithmically extracted before being checked for completeness and coded for statistical analysis. At our institution, reverse transcriptase polymerase chain reaction for SARS-CoV-2 is performed on nasopharyngeal specimens. Test results were obtained from the National Health Laboratory Service (NHLS) TrakCare Web Results Viewer.

Data analysis was performed using VassarStats (available at http://vassarstats.net). Statistical significance was set at *p* < 0.05 and a 95% confidence interval (95% CI) was used.

### Ethical considerations

Ethical approval to conduct this study was obtained from the Health Research Ethics Committee of Stellenbosch University (Reference no. N20/04/002_COVID-19).

## Results

This study included 799 cases. The mean (standard deviation [SD]) age of clients was 39.7 (10.9) years and the majority (619, 77.47%) of clients were women. Most clients were from Tygerberg Hospital (703, 87.98%) and 349 (43.68%) were nurses. Amongst nurses, 326 (93.41%) were women. Other groups included 150 (18.77%) administrative staff members, 63 (7.88%) doctors and 58 (7.26%) general assistants. Most clients were healthy, with 560 (70.09%) recording no comorbidities. The most common comorbidities were asthma (69, 8.64%), diabetes mellitus (61, 7.63%) and obesity (50, 6.26%). Hypertension was excluded from reporting because of a recording error. Table 1 presents the baseline characteristics.

**TABLE 1:** Baseline characteristics.

Characteristic (*N* = 799)	Frequency	Proportion	95% CI
**Sex**			
Female	619	77.47	74.45–80.23
**Institution**			
Tygerberg Hospital	703	87.98	85.54–90.06
Other medical	32	4.01	2.86–5.60
Non-medical	21	2.63	1.73–3.99
Unallocated	43	5.38	4.02–7.24
**Role**			
Nurse	349	43.68	40.28–47.14
Administrative staff	150	18.77	16.21–21.62
Doctor	63	7.88	6.21–9.96
General assistant	58	7.26	5.66–9.27
Porter	18	2.25	1.43–3.53
Allied health	10	1.25	0.68–2.29
Radiology	8	1.00	0.51–1.96
Security	4	0.50	0.19–1.28
Laboratory	1	0.13	0.01–0.81
Other or unallocated	138	17.27	14.81–20.05
**Co-morbidities**			
Asthma	69	8.64	6.88–10.79
Diabetes mellitus	61	7.63	5.99–9.68
Obesity	50	6.26	4.78–8.16
Cardiac disease	39	4.88	3.59–6.60
Previous history of tuberculosis	33	4.13	2.96–5.74
HIV positive	17	2.13	1.33–3.38
On antiretrovirals	23	2.88	1.88–4.28
Pregnant	12	1.50	0.86–2.60
Chronic kidney disease	9	1.13	0.60–2.13
Other lung diseases	9	1.13	0.60–2.13
Chronic liver disease	6	0.75	0.34–1.63
Current tuberculosis	4	0.50	0.19–1.28
None	560	70.09	66.83–73.16

HIV, human immunodeficiency virus; CI, confidence interval.

The median (interquartile range [IQR]) number of symptoms reported was 2 (1–4). The median (IQR) duration of symptoms prior to pre-registration was 4 (2–7) days. This duration was shorter for doctors, at 1.5 (1–4) days. The most common symptom was headache, which was reported by 435 (54.44%) clients, followed by sore throat (361, 45.18%), cough (326, 40.80%) and myalgia (196, 24.53%). The frequency of other symptoms is shown in Table 2. The total number of asymptomatic clients was 145 (18.15%).

**TABLE 2:** Frequency of symptoms amongst all clients pre-registering; and proportion of positive tests for severe acute respiratory syndrome coronavirus 2 according to the number of clients with a valid test result reporting the symptom.

Symptom (*N* = 799)	Symptom frequency	Valid result	Positive result	Proportion	95% CI
Fever	72	61	24	39.34	28.06–51.88
Altered smell	70	50	19	38.00	25.86–51.85
Altered taste sensation	92	65	23	35.38	24.87–47.52
Chills	75	60	18	30.00	19.90–42.51
History of fever	68	50	14	28.00	16.67–42.81
General body weakness	107	84	21	25.00	16.47–35.85
Cough	326	245	56	22.86	18.04–28.51
Myalgia	196	150	32	21.33	15.53–28.56
Headache	435	345	67	19.42	15.59–23.92
Nausea and vomiting	52	39	7	17.95	8.10–34.11
Shortness of breath	115	89	14	15.73	9.61–24.69
Sore throat	361	285	44	15.44	11.71–20.09
Diarrhoea	54	43	5	11.63	5.07–24.48
Irritability	23	12	1	8.33	1.49–35.38
Asymptomatic	145	99	12	12.12	7.07–20.00

CI, confidence interval.

There were 584 (73.09%) clients reporting contact with a confirmed or presumed COVID-19 case. This was self-reported and not verified. Of these, 472 (80.82%) were also symptomatic. Thirty-three (4.13%) clients reported no symptoms or contact and had no indication to test according to pre-registration data, but 14 were still tested.

We found 105 positive results, representing 13.14% (95% CI: 10.97–15.66) of clients using the pre-registration service and 17.77% (95% CI: 14.9–21.06) of those with a valid positive or negative test result. This proportion was the highest amongst nurses (53, 15.19%) compared with six (9.52%) doctors, five (8.62%) general assistants and 12 (8.00%) administrative staff members. There were 486 (60.83%) negative tests, eight (1.00%) indeterminate, nine (1.13%) not run at the laboratory and 191 (23.90%) clients who pre-registered but were not tested.

Fever, altered smell, altered taste sensation and chills were the symptoms with the highest predictive value for SARS-CoV-2 infection, as shown in Table 2. Diarrhoea and irritability had the lowest predictive value, with <10% of these clients testing positive. Twelve (8.28%) asymptomatic clients tested positive. This included three of 33 clients with no reported symptom or contact.

There was a strong association between a greater number of symptoms and a positive test (*p* = 0.0002). Clients with a positive test had a mean (SD) of 3.3 (0.4) symptoms compared with 2.5 (0.2) symptoms amongst clients with a negative test. We particularly noted an increased likelihood of a positive test in patients with 3 or 4+ symptoms (18.55% and 20.70%, respectively) compared with one or two symptoms (8.57% and 6.75%, respectively). When comparing clients presenting rapidly within the first 2 days of symptom onset to those with delayed presentation, there was no significant difference in the number of symptoms (*z*-score = 1.04, *p* = 0.3) or proportion with positive results (*p* = 0.9).

We also noted an increased likelihood of coronavirus infection in patients who had a 2–4- or 5–10-day duration of symptoms (17.70% and 16.95%, respectively) compared with 0–1, 11–15, and 16+ days (9.45%, 11.11%, and 7.41%, respectively).

## Discussion

Streamlined staff testing is a critical component of a broader strategy to maximise the available frontline workforce in the face of the COVID-19 pandemic by decreasing absence.^5^ At our institution, six in seven staff members tested negative for SARS-CoV-2 and were able to return to work earlier than they might otherwise have been able. The remainder were detected and isolated, preventing further spread.

Rapid and sustained uptake of the online pre-registration system shows that this format is acceptable to staff. It allows ease of access, decreases waiting times and expedites test results through prioritisation of staff samples. During the period under review, unpublished departmental data showed that over 5800 tests were performed at our TATC. Implementation of the online pre-registration form therefore resulted in a 14% reduction in the inline clerical work of opening folders whilst clients waited. Its use for clusters from other medical institutions and non-medical facilities demonstrates the potential for further expansion of the system.

Clients presenting via the pre-registration system were young with few comorbidities. It is unclear whether this is reflective of the broader staff population at this academic institution. Alternatively, it could indicate that risk assessment activities performed by the OHD had successfully shielded older, more at-risk individuals from exposure; or that older staff members preferred not to use this hi-tech option. We were able to explain the large proportion of female clients in the study because of a large contingent of nursing staff testing.

Four staff groups were predominantly represented in the data: nurses, administrative staff, doctors and general assistants. This reflects staff members with the most direct contact with patients, but other groups may be being missed. Whilst doctors presented quickly, nurses, administrative staff and general assistants waited longer. This delay represents a period of time staff remain at work and are potentially highly infectious.^6^ The lack of clinical difference between those testing early and late suggests that factors other than the disease process caused the delays. Whilst it was beyond the scope of the current study to identify specific barriers to testing, the authors hypothesise that these include fear of a positive result with potential health implications and stigma, real or imagined pressure to continue working rather than testing and isolating in the context of existing healthcare worker shortages, lack of knowledge regarding how to access testing, non-availability of after-hours testing and bureaucratic delays.^7^ Further studies are needed to determine the effect of these and other barriers.

A large proportion of staff pre-registered but never tested whilst a minority had laboratory issues but were not re-tested. This indicates a significant loss to follow-up, but is in line with the broader experience at our facility precipitated by the massive testing loads as the pandemic progressed and is not specific to the pre-registration system. It is unclear if these potentially infected individuals continued to work without ever testing or continued to isolate for the full 14 days. Integration with a scaled-up OHD will combat this in future and enhance surveillance and protection of staff and patients.^8^

We noted altered smell and altered taste sensation together with flu-like symptoms to be most predictive symptoms of SARS-CoV-2 infection. This reflects that findings noted globally in mildly symptomatic outpatients apply to our local population and shows that they can be incorporated into local guidelines.^9^

Over 8% of asymptomatic individuals, including several without a specific contact, tested positive for SARS-CoV-2. This has also been seen in other healthcare worker screening programmes.^10,11^ Many such asymptomatic and pre-symptomatic individuals may remain undetected as a vector for nosocomial spread to other staff and vulnerable patients.^12^ In a system reliant on self-presentation, asymptomatic and oligo-symptomatic individuals will inevitably be missed. Changes to the provincial community screening and testing strategy advising most symptomatic individuals to self-isolate without testing have significantly reduced the number of tests being performed at the TATC and an alternative surveillance system testing all staff at intervals may now be feasible.^13^

It remains an unanswered question whether future staff testing and isolation strategies can exclude sufficiently low-risk symptomatic presentations, allowing staff to remain at work with suitable precautions in place whilst awaiting a result. Such a strategy could reduce unnecessary isolation and workforce depletion. In this study, no symptom could completely eliminate the risk, whilst asymptomatic infection is discussed above. We found it preferable to focus on rapid turnaround of results.

Limitations of this study are its retrospective nature and the reliance on client self-reported clinical information.

## Conclusion

Staff coronavirus testing using an online pre-registration form is a viable and acceptable strategy. Whilst it requires a degree of technical know-how to set up, it can be inexpensively implemented using free or low-cost third-party services. Benefits include streamlined testing, reduced clerical workload and errors, ease of data capturing and decreased risk of cross-infection. This strategy can replace or complement existing on-site systems.

The primary challenges that need to be overcome for a successful staff testing programme are following up non-attendance and ensuring a rapid turnaround of test results. Institutions also need to ensure that staff members test timely.

Hospital staff remain at high risk for SARS-CoV-2 infection and once infected can become vectors for institutional outbreaks. Current guidelines provide advice on when to test and isolate staff who are symptomatic or have high- or low-risk contacts. These should be continuously updated as we learn more about the virus taking a balanced view to protect staff and maintain the available workforce. Staff testing must also be seen as one component of a larger strategy aimed at protecting staff members, such as wearing masks and face shields, regular handwashing, buddy screening, physical distancing, availability of PPE and special dispensation for COVID-19-related leave.
